# Bonding Performance, Mass Stability and Mechanical Properties of Self-Adhesive Resin Composites and Conventional Dental Composites: A Comparative Laboratory Evaluation

**DOI:** 10.3390/polym18172132

**Published:** 2026-09-01

**Authors:** Md Sofiqul Islam, Vivek Padmanabhan, Nansi Fadi Wahbeh, Lana Yassin Almaghribi, Nada Tawfig Hashim, Smriti Aryal A C, Upoma Guha, Muhammed Mustahsen Rahman

**Affiliations:** 1 Department of Operative Dentistry, RAK College of Dental Sciences, RAK Medical and Health Sciences University, Ras Al-Khaimah P.O. Box 12973, United Arab Emirates; nancy3fadi3wahbeh3@gmail.com (N.F.W.); lasyrna@gmail.com (L.Y.A.); 2 Department of Pediatric Dentistry, RAK College of Dental Sciences, RAK Medical and Health Sciences University, Ras Al-Khaimah P.O. Box 12973, United Arab Emirates; vivek.padmanabhan@rakmhsu.ac.ae; 3 Department of Periodontology, RAK College of Dental Sciences, RAK Medical and Health Sciences University, Ras Al-Khaimah P.O. Box 12973, United Arab Emirates; nada.tawfig@rakmhsu.ac.ae (N.T.H.); mustahsen@rakmhsu.ac.ae (M.M.R.); 4 Department of Oral and Craniofacial Health Sciences, College of Dental Medicine, University of Sharjah, Sharjah P.O. Box 27272, United Arab Emirates; saryalac@sharjah.ac.ae; 5 Department of Adult Restorative Dentistry, College of Dentistry, University of Nebraska Medical Center, 4000 East Campus Loop South, Lincoln, NE 68583-0740, USA; uguha@unmc.edu

**Keywords:** self-adhesive resin composite, universal adhesive system, resin composite, shear bond strength, water sorption, solubility, Vickers hardness, elastic modulus, creep

## Abstract

Self-adhesive resin composites (SARCs) are gaining popularity due to their simplified clinical steps. The objectives of the study were to evaluate the bonding performance, mass stability and mechanical properties of three SARC materials and to compare them with those of two conventional RC materials using a universal adhesive system (UAS). **Materials and Methods:** A total of 80 bovine enamel blocks with 5 mm × 5 mm dimensions were prepared and embedded in acrylic molds. The surface of the enamel was polished using 600-grit carbide to create the smear layer. After acid etching, 2 mm × 2 mm cylinder-shaped restorations were made either with one of the three SARCs or with two types of RCs using UASs. Shear bond strength tests were performed after 24 h of water incubation and after 1 year of artificial aging. The mass stability of the hexagonal-shaped SARC and conventional RC was evaluated using water sorption and solubility tests. Then the mechanical properties of the bonded composites were tested, including Vickers hardness, elastic modulus and creep, after 24 h of water incubation and after 1 year of artificial aging. **Results:** Both the material types and aging showed a significant effect on the bonding performance, mass stability and mechanical properties of SARCs and conventional RCs (*p* < 0.05). The shear bond strength of SARCs was comparable to that of a conventional RC + UAS. Conventional RCs showed lower water sorption and solubility compared to SARCs. The Vickers hardness, elastic modulus and creep properties of some SARCs were inferior compared to conventional RCs both at 24 h and at 1 year. **Conclusions:** The bonding performance of SARCs can substitute the conventional RC+ UAS; however, its inferior mass stability and mechanical properties limit its use in routine dental restorative procedures.

## 1. Introduction

Direct composite restoration is a routine dental procedure performed to restore the esthetics and function of damaged tooth structure. The superior esthetic properties of resin composites (RCs) have made it a preferred material for direct tooth restoration [1]. However, achieving an esthetically satisfactory and functionally acceptable restoration requires a technique-sensitive procedure involving multiple steps such as etching, rinsing, drying, priming, bonding and composite layering. The successful execution of these steps may be compromised by inadequate moisture control, prolonged procedural time, and operator fatigue [2]. Additionally, post-operative complications such as marginal discoloration, post-operative sensitivity, and early loss of retention may occur. The discrepancy between the expected performance of adhesive restorations and their clinical longevity has driven a continued search for materials and protocols that ensure their durability [3].

The conceptual roots of modern adhesive dentistry can be traced back to Professor Buonocore’s ground-breaking observation in 1955 that phosphoric acid etching could enhance the adhesion of resin materials to enamel. The discovery of micro-mechanical retention through resin infiltration into etched enamel prisms revolutionized restorative dentistry [4]. Over the past seven decades, adhesive systems have evolved through multiple generations based on this concept, with dentin bonding agents specifically designed to address the inherently moist, heterogeneous, and biologically active nature of the dentin substrate [5].

The multi-step etch-and-rinse systems have demonstrated well-established bond strength and durability, particularly when bonding to enamel. However, the prolonged chairside procedure and technique sensitivity associated with etch-and-rinse systems led to the development of simplified self-etch adhesives, which used acidic primers to simultaneously condition and prime the tooth substrates. Although the self-etch systems can reduce postoperative sensitivity and operator-related variability, concerns remain regarding their ability to produce durable enamel bonding, particularly because mild self-etch formulations may not adequately etch enamel [6].

Self-adhesive resin composites (SARCs) have been introduced to simplify the restorative workflow by eliminating separate adhesive steps. The SARCs are formulated with acidic functional monomers, often phosphoric acid or carboxylic acid derivatives, intended to partially demineralize the tooth surface and establish chemical interactions with hydroxyapatite. In theory, this dual action allows for micromechanical interlocking and chemical bonding without separate surface pretreatment. Water is typically incorporated into the formulation to facilitate ionization of acidic monomers, enabling interaction with the tooth substrate [7,8]. However, this simplification may involve compromises in material performance, and whether SARCs can provide bonding and mechanical performance comparable to conventional RCs used with contemporary universal adhesive systems (UASs) remains a matter of debate [9].

Several in vitro investigations have reported that conventional RCs used with phosphoric acid etching and modern adhesives consistently achieve high bond strengths to enamel [10,11]. In contrast, SARCs tend to exhibit lower bond strengths, although the reported value may remain above thresholds commonly considered clinically acceptable [12]. The interpretation of these findings is not straightforward. Lower bond strength does not necessarily equate to clinical failure, particularly in low-stress situations or when enamel margins are well supported. Conversely, marginal degradation and microleakage may occur even when initial bond strength appears adequate [13]. Over the past decade, research on self-adhesive RCs has increased steadily; however, the available evidence remains fragmented. Moreover, fewer investigations integrate mechanical properties with bonding performance in a way that reflects the multifactorial demands placed on restorative materials in vivo. One factor that may contribute to variations in bonding performance is the compositional diversity among commercially available SARCs [14].

Available scientific evidence indicates that SARCs offer procedural simplification; however, questions remain regarding their bonding efficacy to enamel and long-term mechanical stability. There is a lack of integrated laboratory studies evaluating both bonding performance and mechanical properties within the same experimental framework. Moreover, comparative studies involving multiple commercially available SARCs and well-established conventional RCs used with a UAS remain limited. Understanding differences in bonding and mechanical performance of these materials is important for informed material selection across different restorative procedures.

The present laboratory investigation was designed to address these research gaps by evaluating the enamel bonding performance, mass stability and key mechanical properties of three SARCs and compare it with those of two conventional RCs in combination with UAS. By evaluating shear bond strength (SBS) to enamel alongside water sorption (WS), solubility (SL), Vickers hardness (HV), elastic modulus (EM), and creep behavior (CR), this study aims to move beyond a single-parameter assessment. This integrated approach was intended to characterize the relationship between bonding performance, mass stability and mechanical properties across different restorative material systems.

The specific objectives of this laboratory study were: (1) To compare the enamel bonding performance of selected SARCs with that of conventional RCs used in conjunction with a UAS as assessed by an SBS. (2) To evaluate and compare the mass stability of these materials to hydrolytic degradation by means of WS and SL tests. (3) To evaluate and compare the mechanical properties of the tested materials by assessing HV, EM and CR tests.

## 2. Materials and Methods

### 2.1. Study Design

The study protocol was reviewed and approved by the research and ethical committee of Ras Al Khaimah Medical and Health Sciences University (RAKCODS-REC-08-2024-25-F) prior to starting the experiment.

### 2.2. Sample Size Calculation

The required sample size was calculated using G*Power statistical software (version 3.1.9.7). The required sample size for the ANOVA was determined using an a priori power analysis. The parameters used for the calculation were an effect size f = 0.5 [15], confidence level of 95%, estimated power (1 − β) = 0.8, numerator degrees of freedom (df) = 10, and number of groups = 10. This analysis indicated that a minimum of 75 specimens were required for the experiment. Accordingly, eight specimens per group (*n* = 8) were used for the SBS experiment. For the mechanical property assessment, the required sample size for repeated-measures ANOVA (within-factors analysis) was determined using a priori power analysis. The parameters used for the calculation were: effect size f = 0.77 [16], confidence level of 95%, estimated power (1 − β) = 0.8, and number of groups = 5. Accordingly, three specimens per group (*n* = 3) were used for the nanoindentation experiment.

### 2.3. Tooth Sample Preparation

Forty freshly extracted bovine incisors were collected and stored in 0.1% thymol solution at 4 °C until use. Prior to including for the experiment, the tooth specimens were examined carefully. Teeth showing visible cracks or unusual discoloration were excluded. The enamel surfaces of the teeth were cleaned with a fluoride-free pumice slurry to remove any debris or biofilm. The teeth were then sectioned to obtain 80 enamel blocks (6 mm × 6 mm) with flat enamel surfaces using a low-speed diamond saw (IsoMet, Buehler, Lake Bluff, IL, USA). The enamel blocks were embedded in 2.5 cm in diameter and 1 cm in thickness using epoxy resin. The enamel surfaces were polished with 600-grit silicon carbide paper to standardize surface roughness and create uniform testing conditions. The enamel surfaces were then etched with 37% phosphoric acid (Scotchbond™ Universal Etchant, 3M, St. Paul, MN, USA) for 20 s and thoroughly rinsed and air-dried using a three-way syringe to remove the smear layer.

### 2.4. Restoration Procedure

The enamel samples were randomly allocated to 5 groups for different restoration procedures. In groups 1 and 2, a universal adhesive (Beauti Bond Xtreme universal adhesive, Shofu Inc., Kyoto, Japan) was applied and polymerized using a light cure (Eighteeth Curing Pen, Changzhou Sifary Medical Technology Co., Ltd., Changzhou City, Jiangsu, China) with a light output of 1500 mW/cm^2^ according to the manufacturer’s instructions. A silicon mold containing a cylindrical opening 2 mm in diameter and 3 mm in height was positioned in the center of the bonded surface and stabilized. RC materials were injected into the mold and polymerized as per the manufacturer’s instructions. In groups 3, 4 and 5, SARCs were injected into the etched enamel surface using the silicon mold and polymerized as per the manufacturer’s instructions. The characteristics of the tested materials in this study are presented in Table 1.

Half of the specimens (8 samples from each group) were stored in distilled water at 37 °C in an incubator for 24 h. The remaining half of the specimens (8 samples from each group) were subjected to artificial aging using an artificial aging device (Thermocycler THE-1100, SDMECHA-TRONIK GMBH, Feldkirchen-Westerham, Munich, Germany). To complete one cycle, the specimens were alternately immersed in water maintained at 55 °C and 5 °C for 30 s each with a dwell time of 5 s. A total of 10,000 thermocycles were performed to simulate approximately 1 year of clinical aging [15].

### 2.5. Bond Strength Test

The bond strengths of conventional RC/UASs and SARCs to enamel were evaluated using shear bond strength (SBS) tests using a universal testing machine (EZ-LX Series, Shimadzu Corporation, Kyoto, Japan). The specimens were mounted in the testing device with the loading blade positioned parallel to and adjacent to the bonded interface. A shear load was applied at a crosshead speed of 1.0 mm/min until bond failure occurred. The shear bond strength value was calculated by using the formula:

Shear Bond Strength (MPa) = Force at Failure (N)/Bonded Area (mm^2^)

After shear bond strength testing, the fractured surfaces of the specimens were examined under a stereomicroscope at 40× magnification to determine the mode of failure. Failure modes were classified as follows: (a) adhesive failure at the adhesive-tooth interface; (b) cohesive failure in enamel; (c) mixed failure (a combination of adhesive and cohesive failures); and (d) cohesive failure in RCs. Composite: failure within the composite material.

### 2.6. Mass Stability Test

A total of 50 RC disks were prepared from the conventional RCs and SARCs (*n* = 10) using a honeycomb-shaped silicon mold (5 mm wide × 2 mm thick). Materials were injected into the mold and covered with a glass slide to obtain a uniform thickness of the materials. The materials were then polymerized according to the manufacturer’s instructions. The initial weight (W1) of each disk was recorded using an analytical balance (BA-E1004, Infitek Co., Ltd., Shanghai, China). The specimens were then immersed in distilled water at 37 °C for 7 days. After immersion, the disks were removed, blotted dry, and weighed again (W2). The water sorption (WS) was calculated using the following equation: WS (µg/mm^3^) = (W2 − W1)/W1. After the WS test, the disks were dried again in a desiccator until a constant weight was achieved. Then the specimens were measured again to determine the final dry mass (W3). The solubility SL was calculated using the following equation: SL (µg/mm^3^) = (W1 − W3)/W1.

### 2.7. Mechanical Properties Test

**Figure 1:**
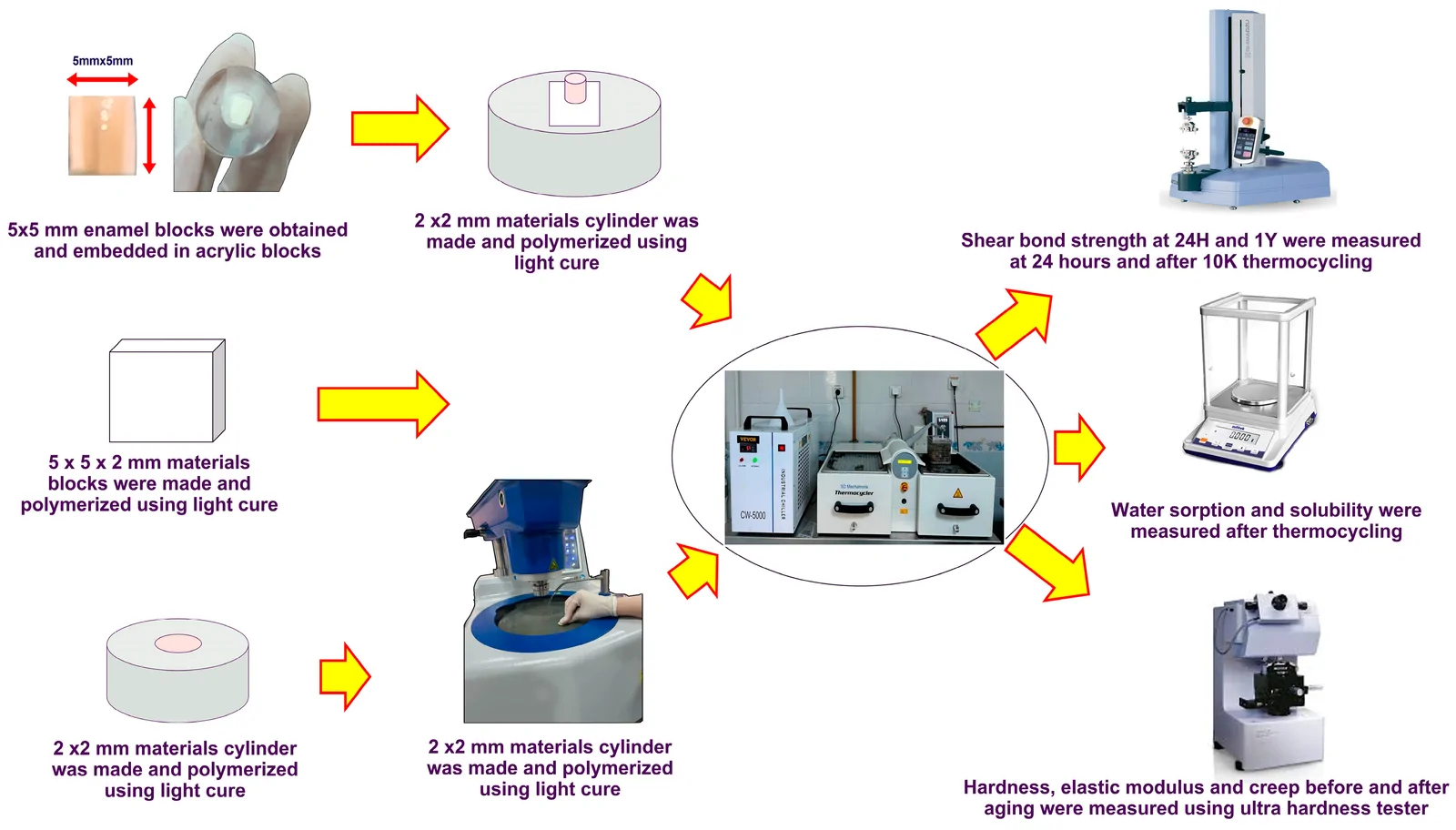
Experiment design.

Conventional RCs and SARCs were placed into 10 mm circular wells measuring 10 mm in diameter and 2 mm in depth of a 2.5 cm circular and 0.6 cm thick acrylic block. The well was covered with a glass slide and pressed gently to remove excess material. The specimens were then polymerized for 40 s. The specimens were then polished with 1000–2500 grit silicon carbide paper in a grinder–polisher (EcoMet 30, Buehler Ltd., Lake Bluff, IL, USA) to obtain a smooth surface. A total of 15 specimens were prepared from the five RC materials (*n* = 3). The specimens were then subjected to mechanical properties testing using a dynamic ultra-micro hardness tester (DUH-211S, Shimadzu Corporation, Kyoto, Japan). An indentation load of 50 mN was applied to the specimen surface using a loading-unloading protocol. A total of 10 indentations were made on each specimen. HV, EM and CR values were determined automatically from each indentation in accordance with ISO 14577-1 (Annex A) [17]. After that, the specimens were allocated for artificial aging using an artificial aging device (Thermocycler THE-1100, SDMECHA-TRONIK GMBH, Feldkirchen-Westerham, Munich, Germany). To complete one cycle, the specimens were subjected to a hot thermal simulation at 55 °C and a cold thermal simulation at 5 °C in a water-submerged condition for 30 s each, with a dwell time of 5 s. In total, 10,000 cycles were conducted to age the specimens by 1 year. Mechanical property testing was subsequently repeated using the ultra-microhardness tester. The experiment design is shown in Figure 1.

### 2.8. Data Analysis

The data were analyzed using statistical software IBM SPSS Statistics, version 24.0 (IBM Corp, Armonk, NW, USA). Descriptive statistics were calculated, and data normality was assessed using the Shapiro–Wilk test. Parametric statistical analyses were performed when the assumptions of normality were satisfied. Two-way ANOVA was conducted to evaluate the effect of the restorative procedure and aging on SBS. For mechanical properties, repeated measures ANOVA was performed to evaluate changes associated with aging. Multiple comparisons among the tested groups were performed using Tukey’s post hoc test. The graphs were generated in GraphPad Prism 10.2.3 (GraphPad Software, Boston, MA, USA).

## 3. Results

**Figure 2:**
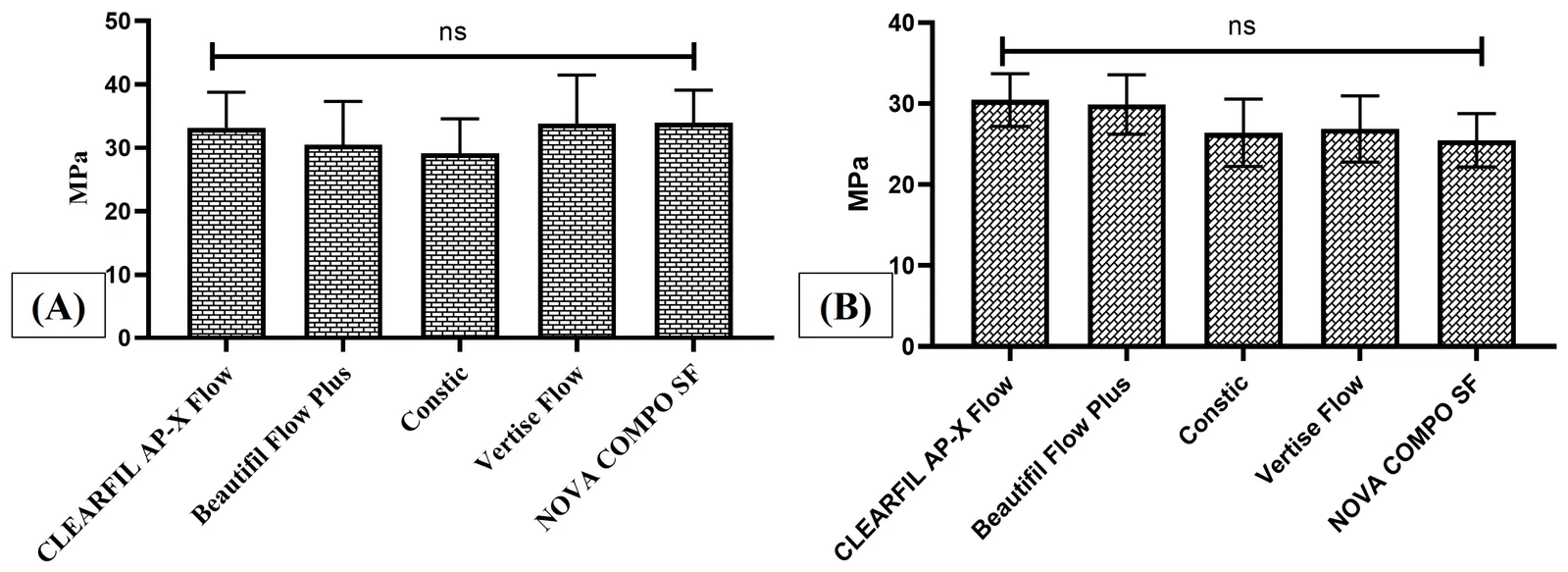
SBS of experimental materials (**A**) at 24 h and (**B**) at 1 year. Groups did not exhibit a statistically significant difference (*p* > 0.05). ns means not significant.

A two-way ANOVA revealed that material type had no significant effect on SBS (*p* = 0.278), whereas aging had a significant effect (*p* = 0.001). The interaction between material type and aging was not statistically significant (*p* = 0.170), with a partial eta-squared effect size of η^2^*p* = 0.087. For the 24 h aged specimens, material type had no statistically significant effect on enamel SBS (*p* = 0.421, η^2^*p* = 0.103). The SBS values of SARCs were comparable to those of a conventional US + RC system. For the 1Y aged specimens, material type had no statistically significant effect on enamel SBS (*p* = 0.078, η^2^*p* = 0.243). The mean SBS values of 24 h and 1Y aged specimens are shown in Figure 2.

**Figure 3:**
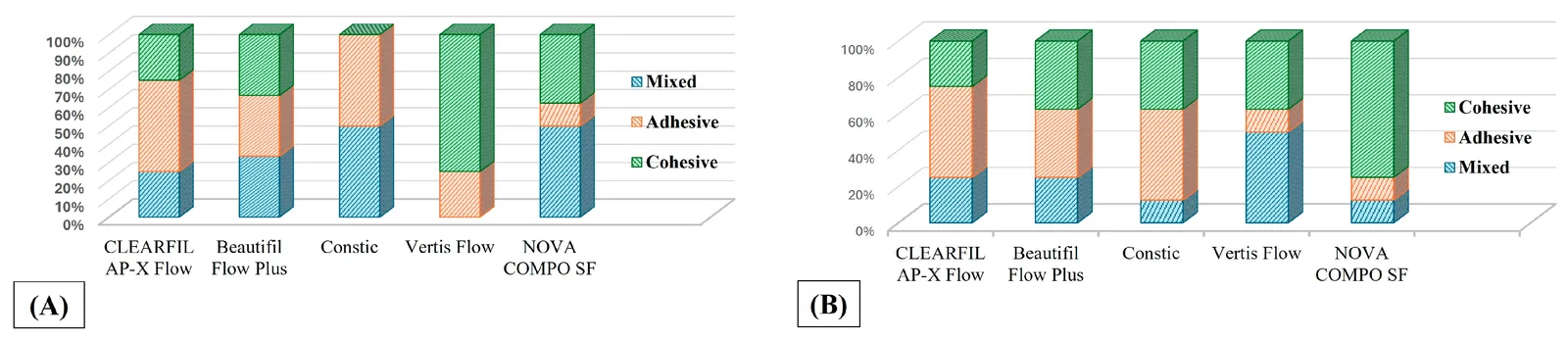
Debond pattern of experimental materials during SBS test (**A**) at 24 h (**B**) at 1 year.

The 24 h and 1-year-aged specimens exhibited similar failure-mode patterns. No notable differences in failure patterns were observed among the tested materials. The failure patterns of the 24 h and 1-year-aged specimens are shown in Figure 3.

**Figure 4:**
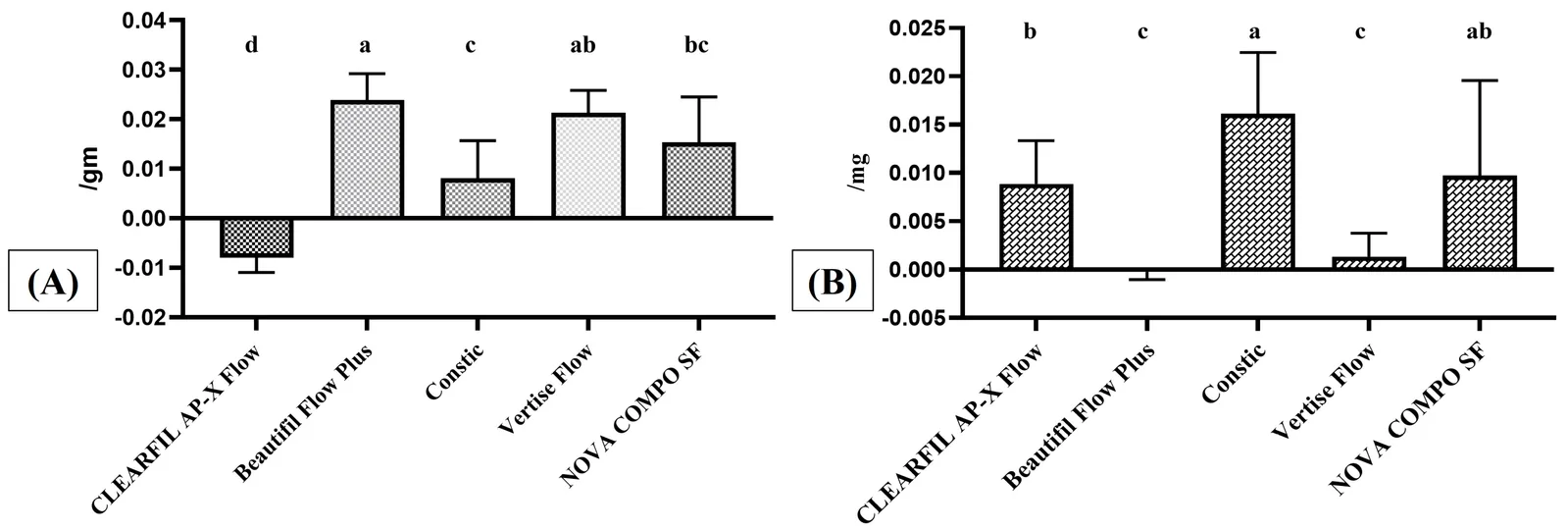
(**A**) WS of experimental materials. (**B**) SL of the experimental materials. Groups denoted by identical letters did not exhibit statistically significant differences (*p* > 0.05).

Unlike SBS, the mass stability parameters differed significantly among the tested materials. One-way ANOVA revealed a significant effect of material type on WS (*p* < 0.0001, η^2^*p* = 0.786). The GIOMER exhibited the highest WS, whereas the micro-hybrid RC exhibited the lowest. Among the SARCs, Constic exhibited the lowest WS, whereas Vertise Flow exhibited the highest. The mean WS for each material is shown in Figure 4A. For SL, the effect of material type was statistically significant (*p* < 0.0001, η^2^*p* = 0.549). Among the SARCs, constic exhibited the highest SL and GIOMER exhibited the lowest. Among the SARCs, Vertise Flow exhibited the lowest SL, which was significantly lower than that of constic and NOVA COMPO SF. The mean SL value of each material is shown in Figure 4B.

**Figure 5:**
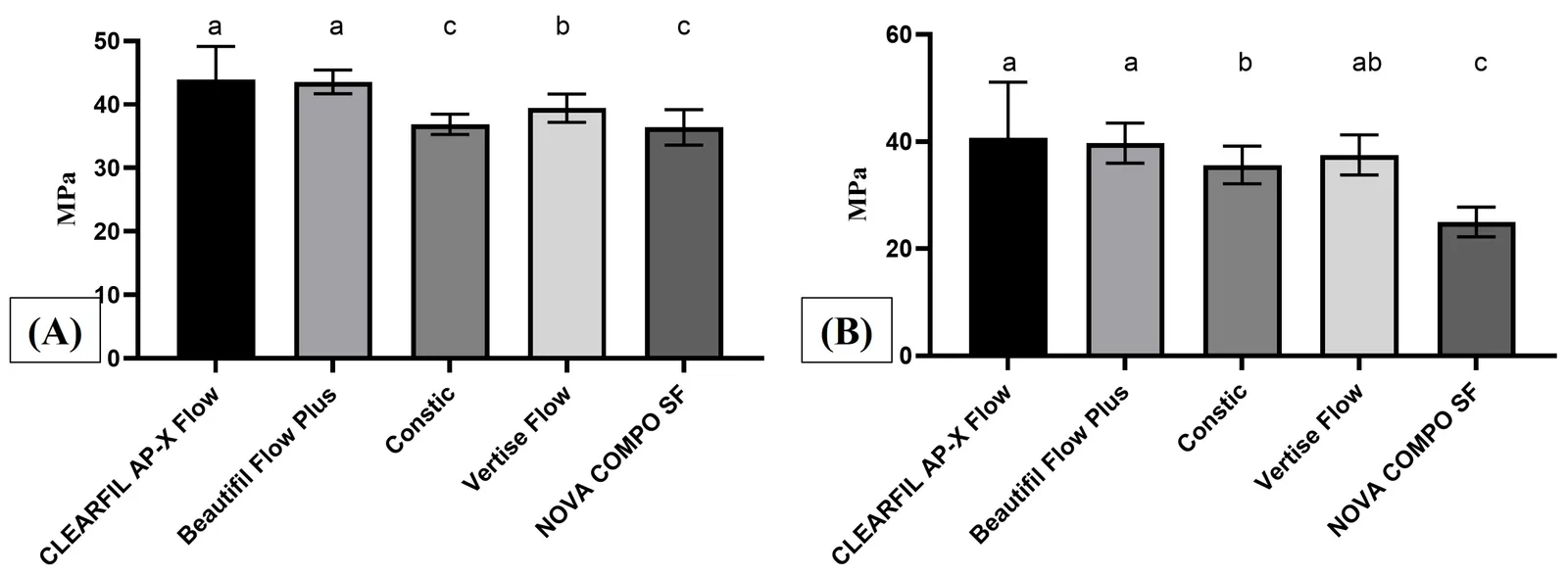
HV of experimental materials (**A**) at 24 h and (**B**) at 1 year. Groups denoted by identical letters did not exhibit statistically significant differences (*p* > 0.05).

Repeated-measures ANOVA revealed a statistically significant effect of aging on HV (*p* < 0.0001, η^2^*p* = 0.362), EM (*p* < 0.0001, η^2^*p* = 0.861), and CR (*p* < 0.0001, η^2^*p* = 0.181). At 24 h, both the micro-hybrid and GIOMER exhibited significantly higher HV values than the SARCs (*p* < 0.05). After 1 year of aging, both the micro-hybrid and GIOMER exhibited significantly higher HV values than SARCs (*p* < 0.05), except for Vertise Flow, which did not differ from those two materials (*p* > 0.05). NOVA COMPO SF exhibited the lowest HV values at both 24 h and after 1 year of aging. The mean HV value for each material in the 24 h and the 1Y group is shown in Figure 5.

**Figure 6:**
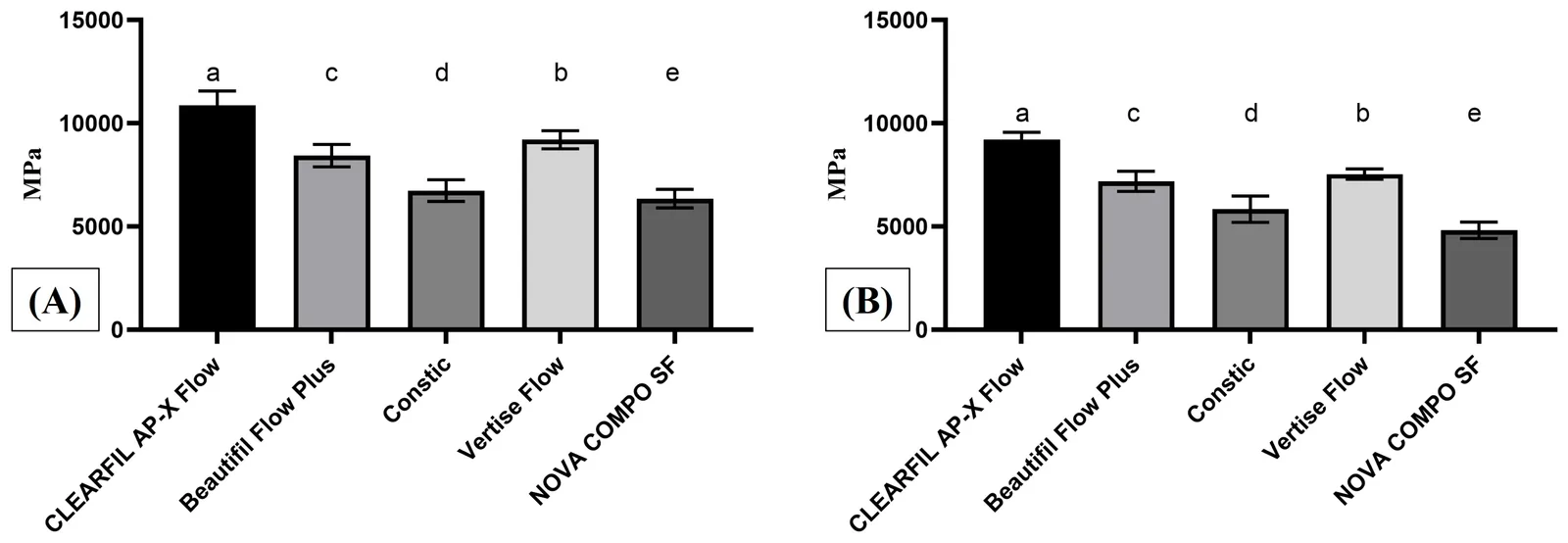
EM of experimental materials (**A**) at 24 h and (**B**) at 1 year. Groups denoted by identical letters did not exhibit statistically significant differences (*p* > 0.05).

Clearfil AP-X Flow exhibited the highest EM, whereas NOVA COMPO SF exhibited the lowest. Significant differences in EM were observed among the tested materials (*p* < 0.05. The mean EM value for each material in 24 h and after 1Y is shown in Figure 6.

**Figure 7:**
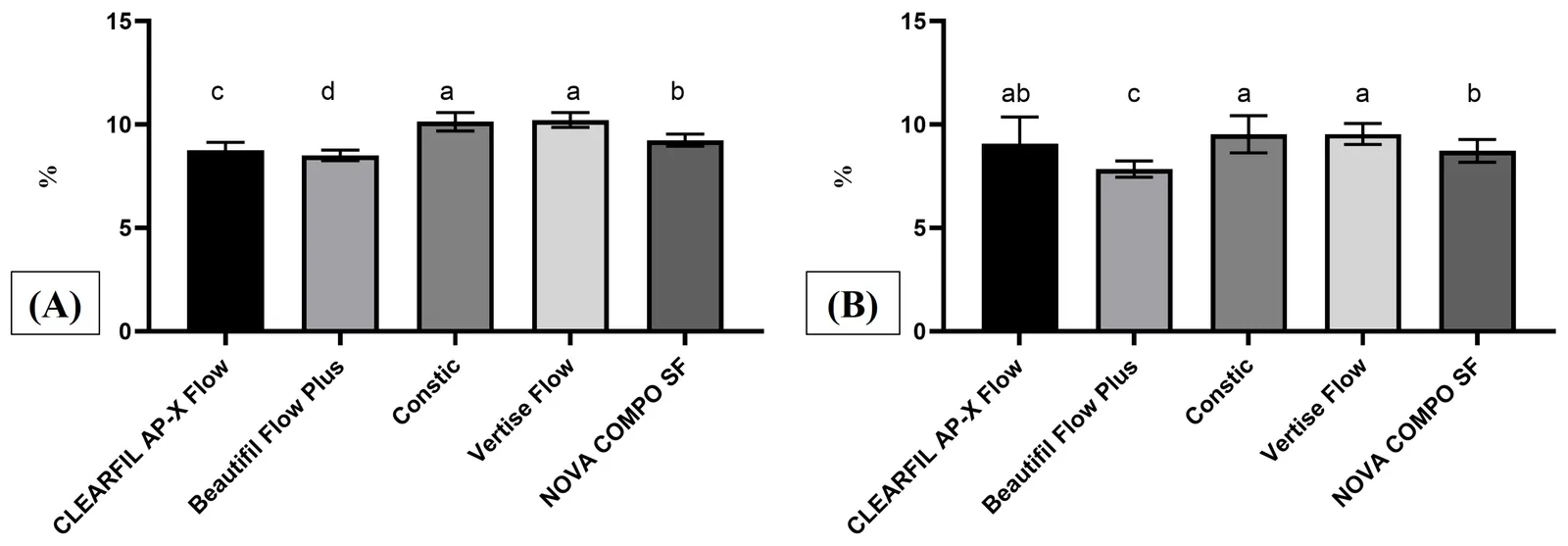
CR of experimental materials (**A**) at 24 h and (**B**) at 1 year. Groups denoted by identical letters did not exhibit statistically significant differences (*p* > 0.05).

For Cr, Vertise Flow exhibited the highest and GIOMER exhibited the lowest value at both 24 h and after 1 year of aging. The mean Cr values for each material at 24 h and 1 year of aging are shown in Figure 7.

## 4. Discussion

The present in vitro study aimed to provide a comprehensive comparison of enamel bonding performance and physical and mechanical behaviors of SARCs and conventional RCs used with a UAS. Unlike previous studies that evaluated only bond strength or individual mechanical parameters, the present study integrated adhesive performance, hydrolytic stability, and mechanical properties within the same experimental framework. This approach provided a broader understanding of the performance of simplified restorative materials under simulated oral conditions.

One of the principal findings of the present study was that the enamel SBS of SARCs was comparable to that of conventional RCs used with a UAS after 24 h of water storage. The absence of a statistically significant difference suggests that, under the conditions of the present study, no detectable difference in initial enamel bonding performance was observed between the tested SARCs and conventional RC/UASs. The comparable initial SBS values observed in the present study may be explained by the acidic functional monomers incorporated into SARC formulations. These monomers are capable of partially demineralizing the enamel surface while simultaneously infiltrating the exposed hydroxyapatite structure. Functional monomers such as glycerol phosphate di-methacrylate or other phosphoric acid derivatives may promote both micromechanical retention and ionic interactions with calcium in hydroxyapatite [18]. However, the etching capacity of self-adhesive composites is generally considered milder than that achieved by 35–37% phosphoric acid. Mild acidic monomers produce a relatively shallow demineralization pattern, which may limit the formation of deep resin tags and reduce the overall micromechanical retention [19]. Previous studies have reported that self-adhesive materials often demonstrate lower enamel bond strength than conventional adhesive systems, particularly when prior phosphoric acid etching is not performed [20]. Notably, in the present study, the enamel surfaces were etched with phosphoric acid before the application of both conventional RCs and SARCs. This pretreatment may have enhanced enamel surface microroughness and improved resin infiltration, thereby reducing potential differences in bonding performance between the two restorative approaches [21].

Artificial aging by thermocycling had a significant effect on SBS, whereas the overall effects of material type and material* aging interaction were not significant. These findings indicate that thermocycling influenced SBS; however, its effect did not differ significantly among the tested material systems. Repeated thermal expansion and contraction of restorative materials and tooth structures during thermocycling may stress the bonded interface, potentially contributing to interfacial degradation [22]. After thermocycling, the SBS value of SARCs remained similar to that of conventional RC/UASs. This finding is consistent with previous reports suggesting that certain SARCs can maintain enamel bond strength following aging, particularly when bonded to pre-etched enamel [23]. However, bond strength represents only one aspect of restorative performance, as factors such as marginal degradation, microleakage, and polymerization shrinkage stresses may also influence long-term restoration integrity [24]. The similar failure patterns observed among the tested materials further indicate that no marked differences in failure behavior occurred between the SARCs and conventional RC/UASs when bonded to etched enamel. A previous investigation by Poitevin et al. in 2013 reported similar findings, with self-adhesive composites exhibiting mixed or cohesive failure modes under certain conditions [20].

In contrast to bond strength results, the hydrolytic stability of the tested materials demonstrated significant variability. WS and SL are critical parameters that reflect the ability of resin-based materials to maintain structural integrity in the moist oral environment. The hydrophilic nature of HEMA and other acidic functional monomers may promote water uptake, potentially resulting in polymer plasticization, filler–matrix debonding, and degradation of silane coupling agents [25]. GIOMER materials contain surface pre-reacted glass (S-PRG) fillers capable of releasing and recharging fluoride ions. Previous research has demonstrated that materials containing ion-releasing glass fillers may exhibit higher water sorption compared with conventional composites due to their ionic and hydrophilic characteristics [26]. Among the SARCs, Constic exhibited the lowest WS, whereas Vertise Flow exhibited the highest. These differences may be attributed to variations in resin matrix composition and filler loading. The SL results did not follow the same pattern as WS across all materials. Constic demonstrated the highest solubility, while GIOMER exhibited the lowest. Solubility reflects the leaching of unreacted monomers or degradation products from the polymer network. Higher SL may be associated with the elution of unreacted components and differences in the degree of polymerization or cross-link density of the resin matrix. Acidic monomers used in SARC formulations may also contribute to increased solubility due to their hydrophilic nature. These findings are consistent with previous reports indicating that SARCs may exhibit higher water sorption and solubility compared with conventional composites because their formulations require hydrophilic components to facilitate self-etching interactions with tooth structure [27].

In the present study, the conventional micro-hybrid RC and GIOMER exhibited significantly higher HV values than most SARCs under both aging conditions. This result can be explained primarily by differences in filler content and resin matrix structure. The higher HV values may be related to differences in filler loading, filler characteristics, and resin matrix composition [28]. Conversely, many self-adhesive composites incorporate lower filler loading and more flexible resin matrices to maintain adequate viscosity and facilitate self-adhesion [29]. Aging significantly affected HV across the tested materials. Thermal cycling can lead to water infiltration and plasticization of the polymer network, reducing surface hardness. Similar reductions in hardness after aging have been reported in previous studies investigating resin composites exposed to water storage and thermal stress [30].

In the present study, Clearfil AP-X Flow exhibited the highest EM, whereas NOVA COMPO SF exhibited the lowest. Materials with higher filler loading generally exhibit higher elastic modulus because rigid inorganic fillers restrict deformation of the polymer matrix. A higher EM may improve resistance to deformation under occlusal loading [31]. However, excessively stiff materials may also concentrate stress at the adhesive interface, potentially increasing the risk of debonding. Conversely, materials with lower modulus values may better absorb stress but could deform more easily under load. Previous studies have emphasized that filler content and filler–matrix interactions are primary determinants of elastic modulus in resin composites [32].

Creep behavior reflects the time-dependent deformation of materials subjected to constant stress. High creep values may compromise marginal integrity and lead to permanent deformation of restorations under occlusal forces. Conversely, lower creep values indicate greater resistance to time-dependent deformation under sustained loading [33]. In the present study, the relatively low creep observed in GIOMER may be related to the reinforcing effect of its filler system. Thermal aging significantly affected CR, which may be associated with water-induced plasticization and changes in the polymer network. Similar findings have been reported in previous investigations evaluating the viscoelastic behaviors of dental composites after water aging [34].

Within the limitations of this in vitro study, the findings suggest that SARCs may provide enamel bonding performance comparable to the tested conventional RC/UAS when phosphoric acid etching is performed. The simplified application procedure of SARCs may offer potential advantages in clinical situations in which reduced procedural complexity is desirable, such as pediatric or minimally invasive restorative procedures. However, the inferior mechanical properties and greater WS or SL observed in some SARCs may raise concerns regarding their performance in areas subjected to high functional stress. Therefore, while SARCs may simplify clinical procedures, careful case selection remains essential. In the present study, the conventional RCs generally demonstrated more favorable mechanical properties than the SARCs; however, the magnitude of these differences varied among tested materials. 

Despite these interesting findings, the generalizability of the present in vitro results to clinical conditions is limited by the absence of factors such as enzymatic degradation, biofilm activity, and cyclic mechanical loading. Additionally, although bovine enamel is widely used as a substitute for human enamel in laboratory bonding studies, differences in substrate characteristics may limit direct extrapolation of the findings to human teeth. Further research should investigate bonding to dentin and evaluate the effects of different enamel pretreatment protocols on the bonding performance of SARCs.

## 5. Conclusions

Within the limitations of this laboratory investigation, SARCs may be considered an alternative to conventional RCs used with UASs in selected clinical situations, such as pediatric dentistry and minimally invasive restorative procedures, where procedural simplicity is advantageous. However, for restoration in high stress-bearing areas or in situations where long-term durability is a primary concern, conventional RCs used with a UAS may remain the preferable option because of their more favorable mechanical performance.

## Figures and Tables

**Table 1:** Characterization of the experimental materials.

Materials	Manufacturer	Key Monomer	Filler Characteristics	LOT Number
CLEARFIL AP-X Flow (Conventional flowable resin composite)	Kuraray Noritake Dental Inc., Tokyo, Japan	TEGDMA	Silanated barium glass 81 wt%	4R0095
BEAUTIFIL Flow Plus (Flowable GIOMER)	Shofu Inc., Kyoto, Japan	Bis-GMA, TEGDMA	Multifunctional glass filler and S-PRG filler 66.8 wt%	042496
Constic (Self-adhesive flowable composite)	DMG, Hamburg, Germany	Bis-GMA, EBADMA, UDMA, HEMA, TEGDMA, HDMA, MDP	Barium-aluminosilicate glass 66 wt%	304995
Vertise Flow (Self-adhesive flowable composite)	Kerr Corp., Orange, CA, USA	GPDM, Bis-EMA, Bis-GMA, UDMA, HEMA and GDM	Barium glass filler, pre-polymerized filler 70 wt%	11641945
Nova Compo SF (Self-adhesive flowable composite)	IMICRYL, Konya, Türkiye	UDMA, Bis-MEPP, TEGDMA	Silicon dioxide and strontium-containing glass 68–69 wt%	L654

## Data Availability

The original contributions presented in this study are included in the article. Further inquiries can be directed to the corresponding author.
